# Effects of Water Cooling on Heat Transfer and Solidification in IN718 Vacuum Arc Remelting

**DOI:** 10.3390/ma19050980

**Published:** 2026-03-03

**Authors:** Zichen Qi, Ming Pan, Panlin Xing, Xujian Jiang, Lvjia Huang, Yukang Jian, Shaowen Lei

**Affiliations:** College of Mechanical Engineering, Zhejiang University of Technology, Hangzhou 310023, China; panming0122@163.com (M.P.); 18709838695@163.com (P.X.); jiangxujian1519@163.com (X.J.); 15870910015@163.com (L.H.); 18840684816@163.com (Y.J.); 19838924016@163.com (S.L.)

**Keywords:** vacuum arc remelting, IN718 alloy, water cooling, heat transfer, solidification microstructure, metallurgical quality

## Abstract

During the vacuum arc remelting (VAR) process, external convective cooling conditions exert a significant influence on both the heat transfer behavior and solidification microstructure of ingots. In this research, Φ 480 mm IN718 alloy VAR ingots were investigated. A heat transfer model for the VAR mold was established based on the equivalent thermal resistance method to analyze the effects of varying external convective cooling conditions on overall heat transfer performance. Industrial-scale VAR experiments were conducted at different cooling water flow velocities (0.48, 0.73 and 1.30 m/s) to assess how external cooling affects molten pool morphology and microstructure evolution. The results indicate that cooling water flow velocity is the primary factor affecting the heat transfer performance of the VAR mold. Increasing the flow velocity significantly enhances radial heat transfer capability while exerting a relatively limited effect on axial heat transfer. Furthermore, as the cooling water flow velocity increases, the molten pool depth decreases markedly, the pool morphology becomes shallower and more symmetric, and the ingot cooling rate is enhanced. Consequently, dendrite coarsening is effectively suppressed, resulting in a significant reduction in secondary dendrite arm spacing. Specifically, when the flow velocity increases from 0.48 to 1.30 m/s, SDAS decreases by 30.4% at the center, 31.0% at R/2, and 26.5% at the edge, and the SDAS-derived equivalent cooling rate (GR) increases from 6.53–18.25 K/min to 19.41–46.01 K/min across the three representative radial locations. A significant enhancement in the metallurgical quality of the VAR ingot is achieved.

## 1. Introduction

Vacuum arc remelting (VAR) is a metallurgical process that refines alloys by remelting them under vacuum conditions. The fundamental principle involves generating heat through a direct-current (DC) arc to melt a consumable electrode. The molten droplets from the electrode tip accumulate to form a molten pool, which subsequently solidifies into an ingot under the forced water cooling of the mold [1,2]. Within the mold, the molten pool undergoes refining and solidification under forced water cooling, thereby improving the microstructure and properties of the ingot [3]. Currently, VAR technology is widely applied in the production of specialty alloys, including superalloys, refractory metals, and ultra-high-strength steels [4]. In recent years, with the increasing demands for dimensional consistency and microstructural homogeneity of nickel-based superalloys in the aerospace industry, VAR processing continues to attract significant attention in both industrial production and academic research. Numerical simulation and experimental studies on large-scale superalloy ingots are being actively pursued [5].

IN718 alloy, also designated as GH4169, is the most widely used superalloy globally, with the most comprehensive range of product types and specifications. Its production volume accounts for approximately half of all wrought superalloys. Due to its excellent strength, oxidation resistance, and corrosion resistance in the temperature range of 650–1000 °C, IN718 alloy is extensively employed in high-temperature applications in the aerospace industry [6]. In recent years, extensive research has been conducted on the fabrication, processing, and service performance of IN718 alloy. The rational design of melting and forming routes is considered a critical prerequisite for achieving high-quality microstructure and stable mechanical properties [7]. In industrial practice, to effectively suppress macrosegregation and metallurgical defects, IN718 alloy is typically processed through multiple melting routes combining vacuum induction melting (VIM) with either electroslag remelting (ESR) or vacuum arc remelting (VAR). Precise control of solidification conditions during remelting is essential for improving ingot internal quality and microstructural homogeneity [8].

During the melting of IN718 alloy, Nb segregation tends to occur in the mushy zone and at the solidification front, leading to metallurgical defects such as freckles, white spots, and inclusion clusters [9]. Previous studies have demonstrated that increasing the cooling rate during VAR can reduce both primary and secondary dendrite arm spacing, refine the microstructure, and suppress Nb segregation and related defect formation [10]. Therefore, investigating the effects of enhanced heat transfer and increased cooling rate on the microstructure evolution of industrial-scale IN718 VAR ingots is of significant importance.

Extensive research has been conducted on the solidification behavior and microstructure evolution of ingots during the VAR process. Existing studies have primarily focused on the relationship between molten pool morphology and solidification microstructure, investigating the effects of pool depth and shape on dendrite growth behavior through both experimental and numerical approaches. Studies have shown that melt rate, arc power, and mold cooling conditions significantly influence pool morphology, solidification front stability, and macrosegregation behavior. Rational control of process parameters is considered one of the key approaches to reducing alloy defects and improving ingot quality [8,11].

Regarding the influence of cooling conditions on microstructure evolution, most existing studies have employed equivalent cooling rates or empirical parameters to characterize cooling intensity [12,13], with limited quantitative correlation between actual process parameters such as external cooling water flow velocity and temperature, overall heat transfer behavior, and ingot microstructural features from the perspective of heat transfer mechanisms. Furthermore, in VAR heat transfer models, external water cooling conditions are typically simplified as constant boundary conditions, and the effects of their variation on individual heat transfer stages and solidification microstructure evolution lack systematic experimental validation [11].

Based on the above research status, this study focuses on Φ 480 mm IN718 alloy VAR ingots. From the perspective of heat transfer mechanisms, the heat transfer behavior during VAR is decomposed into multiple stages, and the effects of cooling water flow velocity, temperature, and specific heat capacity on overall heat transfer are systematically analyzed. On this basis, VAR experiments under different external cooling water flow velocities were designed to investigate the effects of flow velocity variation on molten pool morphology, dendrite distribution, and solidification characteristics. The objective is to provide guidance for the optimization of cooling conditions and rational determination of process parameters in industrial VAR processes.

## 2. Experimental Materials and Methods

### 2.1. Alloy and Electrode Preparation

The IN718 alloy electrodes used in this study were prepared by vacuum induction melting (VIM) using a 12-ton vacuum induction furnace. The chemical composition of the alloy is presented in Table 1, with all major elements falling within the standard composition range for IN718 alloy. To ensure the reliability of experimental comparisons, all electrodes were sourced from the same heat and had identical geometrical dimensions and were subsequently used for the vacuum arc remelting (VAR) experiments.

As shown in Table 1, the measured composition of the electrode falls within the nominal range of IN718 (UNS N07718). All remelting trials (including the two furnace configurations) were conducted using electrodes from the same material batch/heat, and no alloy additions were made during VAR; therefore, the differences discussed in this work are primarily attributed to cooling water conditions rather than bulk chemistry variations.

### 2.2. VAR Equipment and Cooling Water System

**Figure 1:**
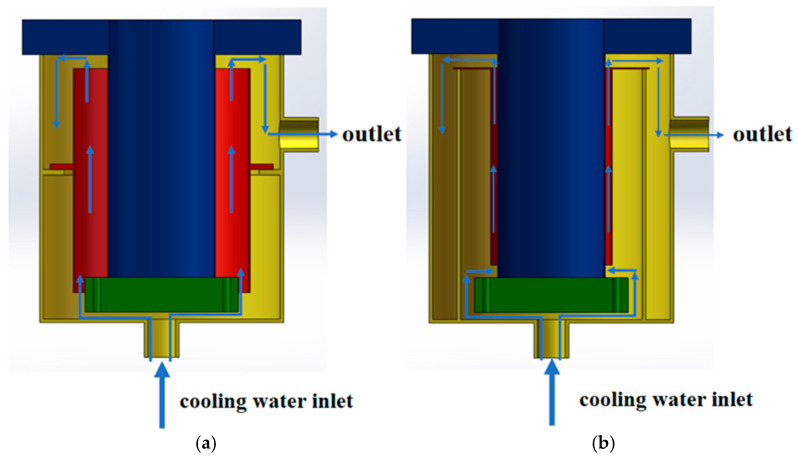
Schematic diagram of VAR furnace structure: (**a**) CONSARC furnace; (**b**) ALD furnace.

Two types of vacuum arc remelting (VAR) furnaces with different structural configurations were employed in this study: CONSARC-type (CONSARC Corp., Rancocas, NJ, USA) and ALD-type (ALD Vacuum Technologies GmbH, Hanau, Germany) VAR furnaces. Both furnaces have a rated capacity of 6 tons, producing ingots with a nominal diameter of Φ 480 mm. As illustrated in Figure 1, the CONSARC furnace features a separate water jacket design with a relatively wide gap between the mold and external water jacket. In contrast, the ALD furnace employs an integrated mold–water jacket structure with a narrower gap. These two configurations exhibit distinct differences in heat transfer paths and heat exchange intensity between the mold and cooling water. Although different VAR furnace configurations were employed, the present study does not attempt to isolate the effects of furnace structure; instead, the discussion is restricted to differences in cooling intensity induced by variations in cooling water flow velocity. During the VAR process, the outer mold wall is subjected to forced cooling by circulating water, which flows upward along the water jacket and is discharged from the top. The cooling water flow velocity is regulated by control valves in the water supply system and monitored in real time by inline flowmeters. In different experimental conditions, external convective heat transfer conditions were adjusted by varying the cooling water flow velocity, while other melting parameters (such as electrode dimensions, melting current, and melt rate) were kept constant to ensure comparability of experimental results.

In an industrial VAR facility, achieving a broad range of cooling water velocities on a single production furnace is not simply a matter of valve adjustment, because the attainable stable velocity window is constrained by the plant cooling circuit (e.g., piping/valve capacity, pump head, and allowable pressure drop) and associated commissioning/safety requirements. Extending the velocity range would typically require hardware-level changes to the cooling loop and downtime and may introduce additional uncontrolled variations in the external convection boundary conditions. Therefore, the three target flow velocity conditions were realized using the existing stable operating windows of the two furnaces while maintaining the same experimental and analytical treatment for comparability.

For transparency, the inlet flow area of the annular water jacket channel is reported as $A_{in}=\frac{\pi }{4}(D_{out}^{2}-D_{in}^{2})$ (where $D_{in}$ is the mold outer diameter and $D_{out}$ is the water jacket inner diameter). The geometric parameters required to calculate $A_{in}$ (i.e., $D_{1}$ and $D_{2}$) are provided in Table 2; therefore, the corresponding volumetric flow rate can be obtained from $Q=vA_{in}$.

The jacket gap width (narrow vs. wide) can influence forced-convection heat transfer by modifying the hydraulic diameter and the flow development/turbulence level in the annular channel, thereby affecting Re, Nu, and the effective convective coefficient h. However, the present study was not designed to isolate the geometry effect at identical volumetric flow rates because the focus is on quantifying the influence of cooling water flow velocity as the primary controllable parameter in the industrial trials. A geometry-isolated assessment (at the same Q) and structural optimization of the water jacket design will be conducted in future work, ideally supported by conjugate CFD/3D transient modeling and pressure drop/flow distribution characterization for equipment design guidance.

### 2.3. Experimental Conditions and Melting Process

In this study, cast electrodes for vacuum arc remelting were first prepared using a 12-ton vacuum induction melting furnace, followed by VAR experiments at different cooling water flow velocities conducted on a 6-ton VAR furnace. To investigate the effects of external cooling water flow velocity on ingot solidification behavior and microstructural characteristics, three groups of VAR experimental conditions were designed. The VAR furnace types and corresponding cooling water flow velocities for each condition are listed in Table 3. Hereinafter, Case 1, Case 2, and Case 3 refer to the three different cooling water flow velocity conditions, and comparative analysis of heat transfer behavior and microstructural characteristics is presented accordingly. The first experiment was conducted on a CONSARC-type VAR furnace at a cooling water flow velocity of 0.48 m/s; the second and third experiments were conducted on an ALD-type VAR furnace at cooling water flow velocities of 0.73 m/s and 1.30 m/s, respectively. Apart from the differences in cooling water flow velocity and furnace configuration, all other melting parameters (including electrode dimensions, melting current, and melt rate) were kept constant. It should be noted that although different VAR furnace configurations were employed in different experimental conditions, this study focuses on the effects of external cooling water flow velocity variation on mold heat transfer behavior and ingot solidification microstructure. The relevant discussions are primarily based on the differences in cooling intensity induced by variations in cooling water flow velocity. After melting, the VAR ingots were sectioned for sampling and metallographic specimen preparation to enable observation and analysis of macrostructure morphology and dendrite characteristics.

### 2.4. Sampling and Microstructure Characterization Methods

To analyze the macroscopic solidification structure and microscopic dendrite characteristics of the VAR ingots, samples were extracted from the ingot top section along the radial direction through the centerline using wire electrical discharge machining (WEDM). The sample dimensions were approximately 480 mm × 350 mm × 40 mm. After grinding and polishing on a large surface grinder, the macrostructure was revealed by etching with a mixed solution of CuSO_4_ (150 g) + H_2_SO_4_ (35 mL) + HCl (500 mL), followed by macroscopic imaging. Microscopic samples were extracted from the edge, mid-radius (R/2), and center positions at the bottom of the macroetched specimen. After standard metallographic preparation, etching was performed using a solution of CuCl_2_ (5 g) + CH_3_COOH (100 mL) + HCl (100 mL). Microstructural observation was conducted using an optical microscope (GX71, Olympus Corporation, Tokyo, Japan) at various magnifications for dendrite morphology analysis. Secondary dendrite arm spacing (SDAS) was measured using the linear intercept method, with multiple representative fields of view selected at each sampling location for statistical analysis. The average values were used for subsequent cooling rate calculations.

## 3. Heat Transfer Model and Parametric Analysis

### 3.1. Heat Transfer Analysis and Model Development for VAR Mold

During the vacuum arc remelting (VAR) process, the solidification behavior and microstructure evolution of the ingot are significantly influenced by overall heat transfer conditions, with external mold cooling conditions being a critical factor controlling cooling intensity. To quantitatively analyze the effects of external cooling water parameter variations on VAR heat transfer behavior, it is necessary to appropriately simplify and model the heat transfer processes associated with the mold.

During the VAR process, the primary heat source is the heat released by arc combustion. The heat absorbed by the inner wall of the copper mold originates from the following sources:

(1) Electrode heat transfer. The arc burns at the electrode tip, where the temperature is highest, and decreases along the electrode length until reaching a stable level. Since convective heat transfer is negligible in a vacuum environment, the electrode dissipates heat primarily through thermal radiation and internal conduction, with most of the heat absorbed by the copper mold and a minor portion removed by the electrode stem and furnace cooling system.
(2) Metal vapor condensation heat release. Metal vapor evaporated from the melting zone predominantly condenses on the cold mold wall surface. The heat released during condensation includes the latent heat of vaporization, latent heat of solidification, and subsequent sensible heat from cooling.
(3) Molten pool radiation. The high-temperature molten pool transfers heat to the surrounding copper mold inner wall primarily through thermal radiation under vacuum conditions.
(4) Liquid metal heat transfer. The molten pool surface temperature exceeds the metal melting point, and natural convection within the pool establishes a temperature gradient distribution. Convective heat transfer occurs between the liquid metal, mold inner wall, and solidified shell, transferring heat to the copper mold.
(5) Ingot solidification heat release. During steady-state melting, the ingot continuously solidifies and releases latent heat of crystallization, which is ultimately transferred to the cooling system through the mold.

During industrial VAR, the melting process generally evolves from an initial transient period (arc ignition and establishment of the molten pool), into a quasi-steady-state melting stage that occupies the majority of the remelting time, and finally into an end-of-melt/hot-top transient when the electrode is nearly consumed and the heat input terminates. In the present work, “steady-state melting” is considered to be reached after the molten pool has been established and the process control has stabilized the operating variables, such that the electrode melt rate and arc power (current/voltage) fluctuate only weakly around their set values, the pool geometry and solidification front evolve slowly in a time-averaged sense, and the imposed boundary conditions (external cooling water flow and helium gap cooling, where applicable) remain constant for a given trial. Under these conditions, the average heat input to the ingot per unit time can be reasonably treated as constant, and the dominant mold heat transfer paths can be analyzed using a steady, one-dimensional equivalent thermal resistance framework; short-term transients such as arc wandering, droplet splashing, and other high-frequency fluctuations are therefore neglected in the model.

Therefore, under steady-state melting conditions with a nominally constant electrode melt rate, the heat input to the system per unit time can be reasonably approximated as constant. Therefore, enhancing the heat dissipation capacity of the mold and increasing the heat removed per unit time can effectively improve the cooling intensity during the VAR process.

The heat dissipation of the mold relies primarily on forced convective heat transfer by external cooling water, as well as convective heat transfer induced by helium introduced into the shrinkage gap between the ingot and mold. As melting progresses and the molten pool position rises, local heat transfer paths and conditions evolve accordingly. To facilitate quantitative analysis of the complex heat transfer process, this study employs the equivalent thermal resistance approach, decomposing the mold-related heat transfer into the following four typical stages [14,15,16]:

(1) Radial heat transfer at the gap without helium; (2) radial heat transfer at the gap with helium; (3) radial heat transfer at the molten pool; (4) axial heat transfer at the molten pool.

To emphasize the effects of external cooling water parameter variations on overall heat transfer behavior while avoiding overly complex numerical calculations, the following reasonable assumptions are made in the modeling process [5,11,17]:

(1) During steady-state melting, the electrode melt rate and heat input per unit time remain constant; (2) each heat transfer process is approximated as one-dimensional steady-state heat transfer and described using equivalent thermal resistance; (3) transient effects such as arc fluctuations and droplet splashing on overall heat transfer behavior are neglected; (4) material thermophysical properties are taken as average values without considering higher-order temperature-dependent effects; (5) the external cooling water flow state is stable, and inlet disturbance effects on heat transfer are neglected.

It should be noted that the present model is intended as an engineering-scale approximation to capture the dominant heat transfer mechanisms and relative trends induced by variations in cooling water parameters, rather than to reproduce the full transient and three-dimensional thermal field of the VAR process.

On this basis, the following sections analyze the effects of cooling water flow velocity, temperature, and specific heat capacity variations on each heat transfer stage and overall heat transfer capability, providing theoretical explanations for the observed changes in ingot solidification microstructure.

### 3.2. Effect of Cooling Water Flow Velocity on VAR Heat Transfer Performance

To quantitatively analyze the effects of external cooling water flow velocity variation on each heat transfer stage and overall heat transfer capability during the VAR process, calculations were performed for cooling water flow velocities ranging from 0.48 to 4.00 m/s while keeping other process parameters constant. The range of 0.48–1.30 m/s covers the cooling water flow velocities used in the present experiments, while higher velocities were included to examine the overall trend of heat transfer coefficient variation with flow velocity. Through equivalent thermal resistance analysis of different heat transfer stages, the effects of cooling water flow velocity variation on local convective heat transfer and overall heat transfer capability were evaluated. The extended flow velocity range is introduced solely for parametric sensitivity analysis and does not imply direct industrial operating conditions.

#### 3.2.1. Effect of Flow Velocity on Radial Heat Transfer at Non-Helium Gap

In the absence of helium between the ingot and mold, the primary heat transfer mode at the gap is radiative heat transfer, and external cooling water participates only in the subsequent convective heat transfer process. Therefore, the effect of cooling water flow velocity variation on overall heat transfer capability in this region is expected to be relatively limited.

**Figure 2:**
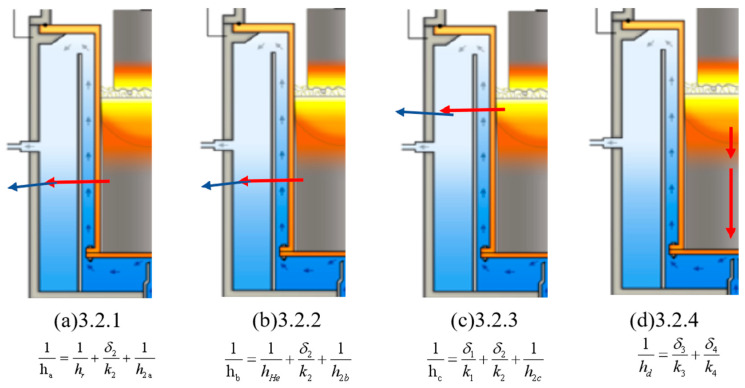
Schematic of the VAR system under steady-state melting and the equivalent thermal resistance representations used in Section 3.2.1, Section 3.2.2, Section 3.2.3 and Section 3.2.4: (**a**) vacuum gap radial heat transfer ($h_{a}$); (**b**) He-filled gap radial heat transfer ($h_{b}$); (**c**) molten pool radial heat transfer including solidified shell conduction ($h_{c}$); and (**d**) molten pool axial heat transfer approximated as one-dimensional conduction ($h_{d}$). Red arrows indicate the dominant heat flow direction in each stage. The color gradient is a schematic illustration for visualization only.

As shown in Figure 2a, the vacuum gap stage is modeled as radial heat transfer through a series network consisting of gap radiation, mold wall conduction, and water-side convection, from which the equivalent coefficient $h_{a}$ is derived. Under non-helium conditions, the radial heat transfer at the copper mold can be simplified into three consecutive stages: heat is first transferred from the ingot to the mold inner wall through vacuum radiative heat transfer across the gap, then conducted through the mold wall to the outer surface, and finally removed by forced convective heat transfer of the external cooling water. This heat transfer process can be approximated as one-dimensional steady-state heat transfer and analyzed using the equivalent thermal resistance method. The overall heat transfer coefficient h_a_ can be expressed as:

(1) $$\frac{1}{h_{a}}=\frac{1}{h_{r}}+\frac{\delta_{2}}{k_{2}}+\frac{1}{h_{2a}}$$

where h_a_ is the overall radial heat transfer coefficient at the non-helium gap, W/(m^2^·K); hᵣ is the radiative heat transfer coefficient from the ingot to the mold inner wall, W/(m^2^·K); δ_2_ is the mold wall thickness, m; k_2_ is the thermal conductivity of the copper mold, taken as 370 W/(m·K); and h_2a_ is the convective heat transfer coefficient of cooling water, W/(m^2^·K).

Since the surface area difference between the ingot gap and mold inner wall is relatively small and the gap dimension is relatively stable, the radiative heat exchange in this region can be approximated as radiative heat transfer between parallel plates. The radiative heat transfer rate Φᵣ can be expressed as [18]:

(2) $$\Phi_{r}=\frac{A\sigma ({T_{1}}^{4}-{T_{2}}^{4})}{\frac{1}{\varepsilon_{1}}+\frac{1}{\varepsilon_{2}}-1}=\frac{A\sigma \Delta T\cdot T_{m}\left(4{T_{m}}^{4}+\Delta T^{2}\right)}{\frac{1}{\varepsilon_{1}}+\frac{1}{\varepsilon_{2}}-1}$$

where A is the surface area at the gap, m^2^; σ is the Stefan–Boltzmann constant, 5.67 × 10^−8^ W/(m^2^·K^4^); T_1_ is the mold inner wall temperature, K; T_2_ is the ingot surface temperature, K; ε_1_ is the emissivity of the mold; ε_2_ is the emissivity of the ingot; Tₘ is the mean of T_1_ and T_2_, K; and ΔT is the temperature difference between T_1_ and T_2_, K.

From Newton’s law of cooling:

(3) $$\Phi_{r}{=h}_{r}A\Delta T$$

Combining Equations (2) and (3), the radiative heat transfer coefficient at the gap can be expressed as:

(4) $$h_{r}=\sigma T_{m}\frac{\left[4T_{m}+{\left(\Delta T\right)}^{2}\right]}{\frac{1}{\varepsilon_{1}}+\frac{1}{\varepsilon_{2}}-1}$$

For the CONSARC furnace production batch with a cooling water flow velocity of 0.48 m/s, the ingot temperature T_1_ is approximately 1523 K; the mold inner wall temperature T_2_ is approximately 573 K; the emissivity of the copper mold ε_1_ is approximately 0.2; and the ingot surface emissivity ε_2_ is approximately 0.4. The calculated radiative heat transfer coefficient hᵣ is approximately 8.29 W/(m^2^·K). The emissivity values were adopted from commonly reported ranges in the literature for copper molds and nickel-based superalloys under high-temperature conditions.

In the external cooling water convective heat transfer process, dimensionless parameters are introduced by non-dimensionalizing the boundary layer momentum equation, including the Nusselt number Nu, Reynolds number Re, and Prandtl number Pr:

(5) $$Nu=\frac{hl}{k}$$

(6) Re=ρvdμ

(7) $$\mathrm{Pr}=\frac{\mu c_{p}}{k}$$

where Nu is the Nusselt number, representing the dimensionless temperature gradient of the fluid at the wall; h is the convective heat transfer coefficient, W/(m^2^·K); l is the flow length, m; k is the fluid thermal conductivity, W/(m·K); Re is the Reynolds number, a measure of the ratio of inertial to viscous forces; ρ is the fluid density, kg/m^3^; μ is the dynamic viscosity, Pa·s; v is the fluid velocity, m/s; d is the hydraulic diameter, m; Pr is the Prandtl number, the ratio of momentum diffusivity to thermal diffusivity; and cₚ is the specific heat capacity at constant pressure, J/(kg·K).

Calculations indicate that the cooling water flow under these conditions satisfies turbulent flow criteria, and the obtained Reynolds and Prandtl numbers fall within the applicable range of the Dittus–Boelter empirical correlation. Therefore, it is reasonable to use this empirical formula to calculate the cooling water convective heat transfer coefficient [19]:

(8) Nu=0.023Re4/5 Prn

where n = 0.4 for heating fluid and n = 0.3 for cooling fluid; the equation is applicable for 0.7 ≤ Pr ≤ 160, Re ≥ 100,000, and L/d ≥ 10.

The hydraulic diameter d for the concentric annular channel is calculated as:

(9) $$d=\frac{4A_{d}}{P}=\frac{4A_{d}}{\pi \left(D_{1}+D_{2}\right)}$$

where A_d_ is the flow cross-sectional area, m^2^; P is the wetted perimeter, m; D_1_ is the mold outer diameter, m; and D_2_ is the water jacket inner diameter, m.

Based on the average cooling water temperature, the water physical properties at atmospheric pressure are as follows: dynamic viscosity μ_a_ ≈ 0.000876 Pa·s, thermal conductivity k_a_ ≈ 0.6 W/(m·K), and specific heat capacity c_a_ ≈ 4.18 × 10^3^ J/(kg·K). The calculated Prandtl number Pr ≈ 6.1, the Reynolds number Re ≈ 7.38 × 10^4^, the hydraulic diameter d = 0.1 m, and L/d = 30, which satisfy the applicable range of the Dittus–Boelter correlation.

Combining Equations (5) and (8), the cooling water convective heat transfer coefficient h_2a_ is:

(10) $$h_{2a}=0.023\frac{k_{a}}{d_{a}}{\left(\frac{\rho_{a}v_{a}d_{a}}{\mu_{a}}\right)}^{0.8}{\left(\frac{\mu_{a}c_{a}}{k_{a}}\right)}^{0.4}$$

Considering inlet effects, a tube length correction factor is introduced, and Equation (10) can be modified as:

(11) $$h_{2a}=0.023\left[1+{\left(\frac{d_{a}}{L}\right)}^{0.7}\right]\frac{k_{a}}{d_{a}}{\left(\frac{\rho_{a}v_{a}d_{a}}{\mu_{a}}\right)}^{0.8}{\left(\frac{\mu_{a}c_{a}}{k_{a}}\right)}^{0.4}$$

The calculated cooling water convective heat transfer coefficient h_2a_ at the non-helium gap is approximately 1839.6 W/(m^2^·K). The thermal resistances of the three stages in this process are shown in Table 4.

Based on the thermal resistance distribution results and calculations of heat transfer coefficient variation with cooling water flow velocity, under non-helium gap conditions, the radiative heat transfer resistance from the ingot to the mold inner wall dominates the overall thermal resistance and represents the limiting stage of this heat transfer process. In comparison, the thermal resistances of mold wall conduction and external cooling water convective heat transfer account for relatively small proportions. The convective heat transfer coefficient of the cooling water, the overall heat transfer coefficient of this section, and their relative improvement obtained by varying the water flow rate are shown in Table 5. As the cooling water flow velocity increases from 0.48 m/s to 1.30 m/s, the overall heat transfer coefficient increases by only approximately 2.4%, indicating that even with enhanced convective heat transfer capability through increased cooling water flow velocity, the improvement in overall heat transfer performance in this region remains relatively limited.

**Figure 3:**
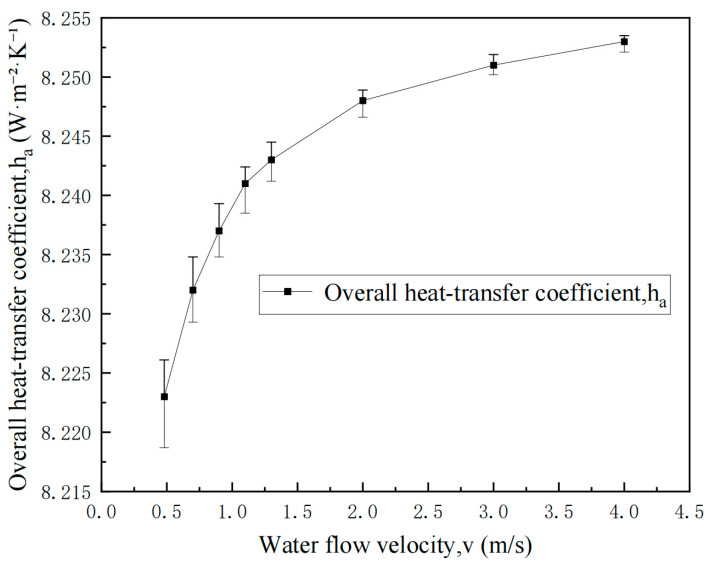
Effect of cooling water flow velocity on the overall heat transfer coefficient $h_{a}$ in the helium-free gap. Error bars: ±10% perturbation in $h_{2a}$ (Dittus–Boelter) propagated through the series thermal resistance model.

To visualize the uncertainty of the calculated $h_{a}$, error bars were obtained by a one-at-a-time perturbation of the water-side convection coefficient $h_{2a}$. Specifically, $h_{2a}$ was calculated using the Dittus–Boelter correlation for turbulent flow and then varied by ±10% to represent the typical scatter of empirical correlations and input property uncertainty. For each flow velocity condition, three deterministic calculations (baseline, $h_{2a}$ × 0.9, and $h_{2a}$ × 1.1) were performed, and the resulting bounds of $h_{a}$ propagated through the series thermal resistance relation are reported as the error bars in Figure 3.

From an industrial energy efficiency perspective, the heat transfer enhancement shows diminishing returns at high velocities because turbulent forced-convection correlations imply h increases sublinearly with V (i.e., $Nu\propto Re^{m}$ with m<1). Consequently, identifying an optimal balance between pumping energy and marginal metallurgical benefit requires plant-specific ΔP–Q and pump efficiency data and is left for future work.

#### 3.2.2. Effect of Flow Velocity on Radial Heat Transfer at Helium-Filled Gap

As shown in Figure 2b, for the He-filled gap stage, the gap term is replaced by the effective helium heat transfer coefficient, while the mold wall conduction and water-side convection terms remain in series, yielding $h_{b}$. After helium is introduced between the ingot and mold, the primary heat transfer mode at the gap transitions from radiative to helium-dominated convective heat transfer, significantly enhancing the overall heat transfer capability at the gap. Under these conditions, the proportion of external cooling water convective heat transfer in the overall thermal resistance increases, and the effect of cooling water flow velocity variation on heat transfer performance is expected to become more pronounced.

After helium is introduced at the gap, the radial heat transfer at the copper mold can be simplified into three consecutive stages: heat is first transferred from the ingot to the mold inner wall through helium convective heat transfer, then conducted through the mold wall to the outer surface, and finally removed by forced convective heat transfer of the external cooling water. Using the equivalent thermal resistance method, the overall heat transfer coefficient h_b_ for this process can be expressed as:

(12) $$\frac{1}{h_{b}}=\frac{1}{h_{He}}+\frac{\delta_{2}}{k_{2}}+\frac{1}{h_{2b}}$$

where h_b_ is the overall radial heat transfer coefficient at the helium-filled gap, W/(m^2^·K); h_He_ is the helium convective heat transfer coefficient at the gap, W/(m^2^·K); and h_2b_ is the cooling water convective heat transfer coefficient for this process, W/(m^2^·K).

The helium cooling stage in this process can be simplified as convective heat transfer between parallel plates. The heat transfer coefficient is calculated as shown in Equation (10):

(13) $$h_{He}=0.023\frac{k_{b}}{d_{b}}{\left(\frac{\rho_{b}v_{b}d_{b}}{\mu_{b}}\right)}^{0.8}{\left(\frac{\mu_{b}c_{b}}{k_{b}}\right)}^{0.4}$$

where k_b_ is the thermal conductivity of helium, taken as 0.25 W/(m·K); d_b_ is the gap width, taken as 0.002 m; ρ_b_ is the helium density, taken as 3.98 kg/m^3^; μ_b_ is the dynamic viscosity of helium, taken as 2.5 × 10^−5^ Pa·s; and c_b_ is the specific heat capacity of helium at constant pressure, taken as 5190 J/(kg·K).

Calculation results indicate that the helium flow state under the selected conditions is stable, and its physical properties and gap dimensions satisfy the applicable conditions for the parallel plate convective heat transfer model. Therefore, it is reasonable to employ the parallel plate convective heat transfer model to estimate the helium convective heat transfer coefficient in engineering-scale modeling. The calculated helium convective heat transfer coefficient h_He_ is approximately 1403.1 W/(m^2^·K).

As shown in Table 6, after helium is introduced at the gap, helium convective heat transfer and external cooling water convective heat transfer together constitute the primary heat transfer resistances, with helium convective heat transfer accounting for approximately 53.83% and cooling water convective heat transfer approximately 41.07%. Both play significant roles in the overall heat transfer process. Compared with non-helium conditions, external cooling water convective heat transfer is no longer a secondary stage, and its enhancement can effectively improve overall heat transfer capability.

**Figure 4:**
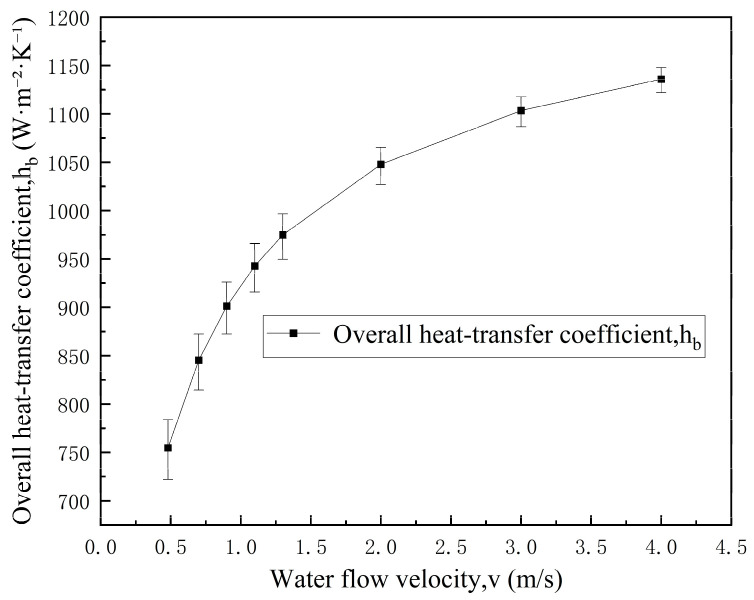
Effect of cooling water flow velocity on the overall heat transfer coefficient $h_{b}$ in the helium-filled gap. Error bars: ±10% perturbation in $h_{2b}$ (Dittus–Boelter) propagated through the series thermal resistance model.

As cooling water flow velocity increases, the cooling water convective heat transfer coefficient increases significantly, thereby notably reducing the corresponding thermal resistance. As shown in Table 7 and Figure 4, when the cooling water flow velocity increases from 0.48 m/s to 1.30 m/s, the overall heat transfer coefficient under helium-filled conditions increases by approximately 29.1%, indicating that radial heat transfer at the gap can be significantly enhanced by increasing cooling water flow velocity under helium-filled conditions.

Comparison between non-helium and helium-filled gap conditions reveals that helium introduction not only significantly reduces the heat transfer resistance at the gap but also enhances the regulatory role of external cooling water convective heat transfer in the overall heat transfer process.

Following the uncertainty treatment described in Section 3.2.1, the error bars in Figure 4 were obtained by applying a ±10% perturbation to the Dittus–Boelter-based $h_{2b}$ and propagating it through the series thermal resistance relation for $h_{b}$.

#### 3.2.3. Effect of Flow Velocity on Radial Heat Transfer at Molten Pool

As shown in Figure 2c, for the molten pool radial stage, an additional conduction term through the solidified shell is included in series with mold wall conduction and water-side convection, leading to $h_{c}$. In the molten pool region, the high temperature of the liquid metal provides a large driving force for heat transfer. Heat is primarily transferred radially through the mold wall to the external cooling water, with external cooling water convective heat transfer dominating the overall heat transfer process. Therefore, the effect of cooling water flow velocity variation on radial heat transfer capability at the molten pool is expected to be most significant.

The radial heat transfer at the molten pool can be simplified as heat transfer from the pool through the solidified shell and mold wall to the mold outer surface, and it is finally removed by forced convective heat transfer of the external cooling water. Using the equivalent thermal resistance method, the overall heat transfer coefficient h_c_ can be expressed as:

(14) $$\frac{1}{h_{c}}=\frac{\delta_{1}}{k_{1}}+\frac{\delta_{2}}{k_{2}}+\frac{1}{h_{2c}}$$

where h_c_ is the overall radial heat transfer coefficient at the molten pool, W/(m^2^·K); k_1_ is the thermal conductivity of the thin solidified shell, taken as 25 W/(m·K); δ_1_ is the solidified shell thickness, taken as 0.005 m; and h_2c_ is the external cooling water convective heat transfer coefficient for this process, W/(m^2^·K). The calculation method for the external cooling water convective heat transfer coefficient h_2c_ and the empirical correlation employed are consistent with those described previously and are not repeated here.

The thermal resistance distribution for radial heat transfer at the molten pool indicates that external cooling water convective heat transfer accounts for the major portion of the overall thermal resistance, representing the limiting stage controlling radial heat transfer capability at the molten pool. Compared with the gap region, the thermal resistances of mold wall conduction and solidified shell conduction account for relatively smaller proportions. Therefore, enhancing cooling water convective heat transfer intensity can effectively reduce the overall thermal resistance and significantly enhance radial heat transfer at the molten pool. The thermal resistance distribution of this section is summarized in Table 8. 

**Figure 5:**
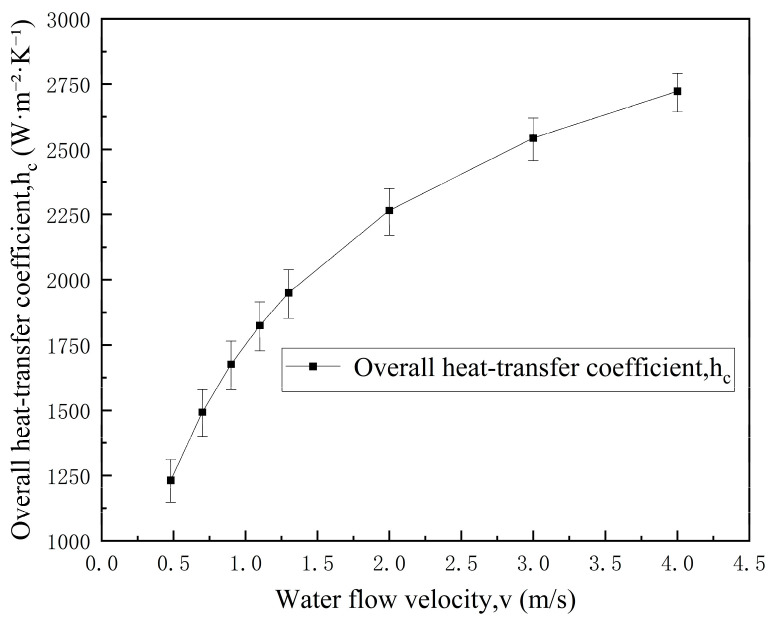
Effect of cooling water flow velocity on the overall heat transfer coefficient $h_{c}$ in the molten-pool region. Error bars: ±10% perturbation in $h_{2c}$ (Dittus–Boelter) propagated through the series thermal resistance model.

As cooling water flow velocity increases, the external cooling water convective heat transfer coefficient increases significantly, leading to a marked reduction in overall thermal resistance at the molten pool. As shown in Table 9 and Figure 5, when the cooling water flow velocity increases from 0.48 m/s to 1.30 m/s, the improvement in overall radial heat transfer coefficient at the molten pool far exceeds that in the gap region, indicating that cooling water flow velocity variation has the most significant enhancement effect on radial heat transfer at the molten pool.

In summary, radial heat transfer at the molten pool is primarily controlled by external cooling water convective heat transfer, making it the heat transfer region most effectively regulated by cooling water flow velocity. This is also one of the important reasons why cooling water parameter variations can significantly affect ingot solidification behavior.

Similarly, the error bars in Figure 5 were constructed by ±10% perturbation in the Dittus–Boelter-based $h_{2c}$ and propagation through the series thermal resistance relation for $h_{c}$, consistent with Section 3.2.1.

#### 3.2.4. Effect of Flow Velocity on Axial Heat Transfer at Molten Pool

As illustrated in Figure 2d, the molten pool axial stage is treated separately and approximated as one-dimensional axial conduction, based on which the equivalent coefficient $h_{d}$ is defined. Unlike radial heat transfer, axial heat transfer at the molten pool occurs primarily along the ingot height direction, characterized by long heat transfer paths and large geometric dimensions, with no direct convective heat transfer interface with the external cooling water. Therefore, the effect of cooling water flow velocity variation on axial heat transfer capability at the molten pool is expected to be relatively limited.

The axial heat transfer at the molten pool can be simplified as heat transfer along the ingot axial direction through the solidified layer and solid ingot to downstream regions. This heat transfer process is primarily controlled by material thermal conductivity and geometric dimensions. Using the equivalent thermal resistance method to analyze axial heat transfer at the molten pool, the axial heat transfer coefficient h_d_ can be expressed as:

(15) $$\frac{1}{h_{d}}=\frac{\delta_{3}}{k_{3}}+\frac{\delta_{4}}{k_{4}}$$

where h_d_ is the overall axial heat transfer coefficient at the molten pool, W/(m^2^·K); δ_3_ is the equivalent axial heat transfer distance from the pool bottom to the mold bottom, m; k_3_ is the thermal conductivity of the ingot, taken as 25 W/(m·K); δ_4_ is the mold bottom thickness, approximately 0.1 m; and k_4_ is the thermal conductivity of the mold bottom, taken as 370 W/(m·K).

In the axial heat transfer process at the molten pool, external cooling water does not directly participate in axial heat transfer but indirectly affects the axial heat transfer driving force by influencing the overall temperature distribution of the mold and ingot. As the casting process progresses, the molten pool gradually solidifies and rises, and the axial heat transfer distance from the pool bottom to the mold bottom continuously increases. The variation in the axial overall heat transfer coefficient during this process is shown in Table 10.

As shown in Table 10, as the molten pool position rises, the axial overall heat transfer coefficient at the molten pool exhibits a significant decreasing trend. This is because axial heat transfer primarily relies on the thermal conductivity of the material itself, and extension of the heat transfer path significantly increases the overall thermal resistance, leading to rapid weakening of heat transfer capability. Under these conditions, the scope for effective enhancement of axial heat transfer through adjustment of external cooling water parameters is relatively limited.

In summary, axial heat transfer at the molten pool is primarily controlled by ingot material thermal conductivity and axial geometric dimensions. External cooling water flow velocity variation only indirectly affects this process by changing the overall temperature field, with regulatory effects significantly weaker than those on radial heat transfer. It should be noted that these conclusions are obtained from a one-dimensional steady-state equivalent thermal resistance model, which captures the average heat transfer response but does not resolve circumferential non-uniformities or transient effects that may drive melt pool asymmetry. A three-dimensional transient heat transfer model would allow for circumferentially resolved, time-dependent temperature/heat flux predictions and, thus, could improve the prediction of melt pool asymmetry and symmetry changes observed experimentally under different flow conditions.

### 3.3. Effect of Cooling Water Temperature on VAR Heat Transfer Performance

For turbulent liquid flow inside tubes, temperature changes primarily cause variations in liquid viscosity, while other property changes are relatively small and can be neglected [18,19]. When the temperature difference between the fluid and tube wall is large and the liquid is being heated, the Sieder–Tate correlation employs (μ_f_/μ_w_)^0.11^ as a correction term for non-uniform thermophysical properties:

(16) Nu=0.023μfμw0.11Re0.8Pr0.4

where μ_f_ is the dynamic viscosity of the fluid at fluid temperature t_f_, N·s/m^2^; μ_w_ is the dynamic viscosity of the fluid at wall temperature t_w_, N·s/m^2^; and applicable conditions are: 0.7 < Pr < 16,700, Re > 10,000, and (l/d) > 10.

At a cooling water flow velocity of 0.48 m/s, the applicable range of this equation is satisfied. Combining with Equation (5), the cooling water convective heat transfer formula with temperature correction can be obtained:

(17) $$h=0.023{\left(\frac{\mu_{f}}{\mu_{w}}\right)}^{0.11}\left[1+{\left(\frac{d}{L}\right)}^{0.7}\right]\frac{k}{d}{\left(\frac{\rho vd}{\mu }\right)}^{0.8}{\left(\frac{\mu c}{k}\right)}^{0.4}$$

**Figure 6:**
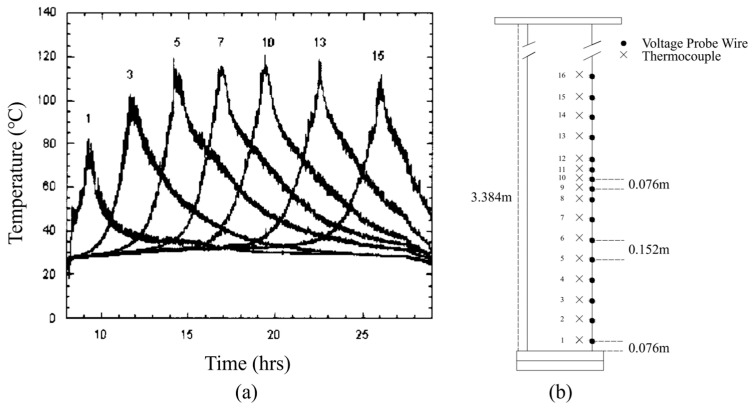
(**a**) Temperature–time profiles at various locations on mold outer wall; (**b**) sensor layout diagram on mold outer wall [21]. The numbers above the curves indicate the positions (IDs) of the thermocouples.

According to relevant research [20], the temperature distribution at different positions on the mold outer wall during the VAR process is shown in Figure 6, providing a basis for wall temperature selection in cooling water temperature correction. The mold outer wall temperature at the molten pool is approximately 110 °C, and at the gap is approximately 70 °C. Cooling effects at water temperatures of 0 °C, 10 °C, 20 °C, and 30 °C were compared. The viscosity coefficients of water at various temperatures are tabulated as follows (Table 11). Based on the preceding thermal resistance analysis, external cooling water convective heat transfer accounts for a relatively small proportion in non-helium gap conditions and axial heat transfer at the molten pool. Therefore, when analyzing the effects of cooling water temperature variation, the focus is on its effect on radial heat transfer, while effects on other heat transfer stages are neglected.

The convective heat transfer coefficient of the cooling water calculated from the water temperature variation, the overall heat transfer coefficient of the whole section, and the heat transfer enhancement are summarized in Table 12. At a cooling water temperature of 30 °C and flow velocity of 0.48 m/s, the calculated convective heat transfer coefficient at the gap is approximately 1982.4 W/(m^2^·K), and that at the molten pool is approximately 2099.2 W/(m^2^·K). Calculation results for temperatures of 20 °C, 10 °C, and 0 °C are shown in the table. When the cooling water temperature decreases from 30 °C to 0 °C, the heat transfer improvement at the helium-filled gap is only 3.06%, and at the molten pool radial heat transfer is only 5.79%. Compared with the effect of flow velocity on heat transfer efficiency, the effect of water temperature is significantly weaker than that of cooling water flow velocity variation on heat transfer performance.

### 3.4. Effect of Cooling Water Specific Heat Capacity on VAR Heat Transfer Performance

In mold cooling enhancement studies, modifying the thermophysical properties of the cooling medium to improve heat transfer capability is a common approach. Among these, adding nanoparticles to fluids to increase specific heat capacity has received considerable attention [21]. However, in mold cooling processes dominated by external convective heat transfer and overall thermal resistance distribution, the actual effect of cooling water specific heat capacity variation on overall heat transfer performance requires quantitative evaluation. Therefore, this section analyzes the effect of cooling water specific heat capacity variation on overall mold heat transfer performance while excluding the effects of flow velocity and temperature variations. To exclude the effects of other parameter variations, cooling water flow velocity and inlet temperature were kept constant, and only cooling water specific heat capacity was varied. Three conditions with specific heat capacities of 4.4 × 10^3^ J/(kg·K), 4.6 × 10^3^ J/(kg·K), and 4.8 × 10^3^ J/(kg·K) were selected for comparative analysis of radial heat transfer at the gap and molten pool. Calculation results are shown in Table 13.

As shown in Table 13, as cooling water specific heat capacity increases, the overall heat transfer coefficients at both the gap and molten pool increase, but the overall improvement is relatively small. When cooling water specific heat capacity increases from 4.18 × 10^3^ J/(kg·K) to 4.8 × 10^3^ J/(kg·K), the overall heat transfer coefficients at the gap and molten pool increase by only 2.30% and 3.81%, respectively.

This is because in the mold cooling process, overall heat transfer capability is primarily controlled by the external convective heat transfer coefficient and the thermal resistance distribution structure of each heat transfer stage. Although changes in cooling water specific heat capacity affect the fluid heat-carrying capacity, they do not change the location of major heat transfer resistances, nor do they significantly improve the convective heat transfer coefficient itself.

Therefore, within the investigated parameter range, the enhancement of overall mold heat transfer performance through increased cooling water specific heat capacity is limited, with its effect significantly weaker than that of cooling water flow velocity variation on heat transfer performance.

### 3.5. Summary of Chapter 3

This chapter systematically analyzed the effects of cooling water parameters on heat transfer performance during the VAR mold cooling process. Based on the equivalent heat transfer model, calculations and comparisons were performed for radial heat transfer at the mold gap and both radial and axial heat transfer at the molten pool, clarifying the controlling mechanisms of different heat transfer directions and stages.

The results indicate that cooling water flow velocity variation has the most significant effect on mold heat transfer performance. As cooling water flow velocity increases, the overall radial heat transfer coefficients at both the mold gap and molten pool increase markedly, indicating that external convective heat transfer is the dominant factor in the radial heat transfer process. In comparison, axial heat transfer at the molten pool is primarily controlled by ingot material thermal conductivity and axial geometric dimensions, with limited enhancement effect from cooling water flow velocity variation.

On this basis, the effects of cooling water temperature and specific heat capacity variations on mold heat transfer performance were further analyzed. The results show that within the investigated parameter range, both cooling water temperature and specific heat capacity variations affect heat transfer performance to some extent, but their effects are significantly weaker than that of cooling water flow velocity variation, and they are unable to alter the major thermal resistance distribution structure in the overall heat transfer process.

In summary, during the VAR mold cooling process, cooling water flow velocity is the key parameter affecting heat transfer performance, while the regulatory effects of cooling water temperature and specific heat capacity are relatively limited. The results of this chapter provide a theoretical basis for subsequent optimization of mold cooling parameters and process control.

## 4. Experimental Results and Discussion

### 4.1. Macrostructure Characteristics of VAR Ingots at Different Cooling Water Flow Velocities

**Figure 7:**
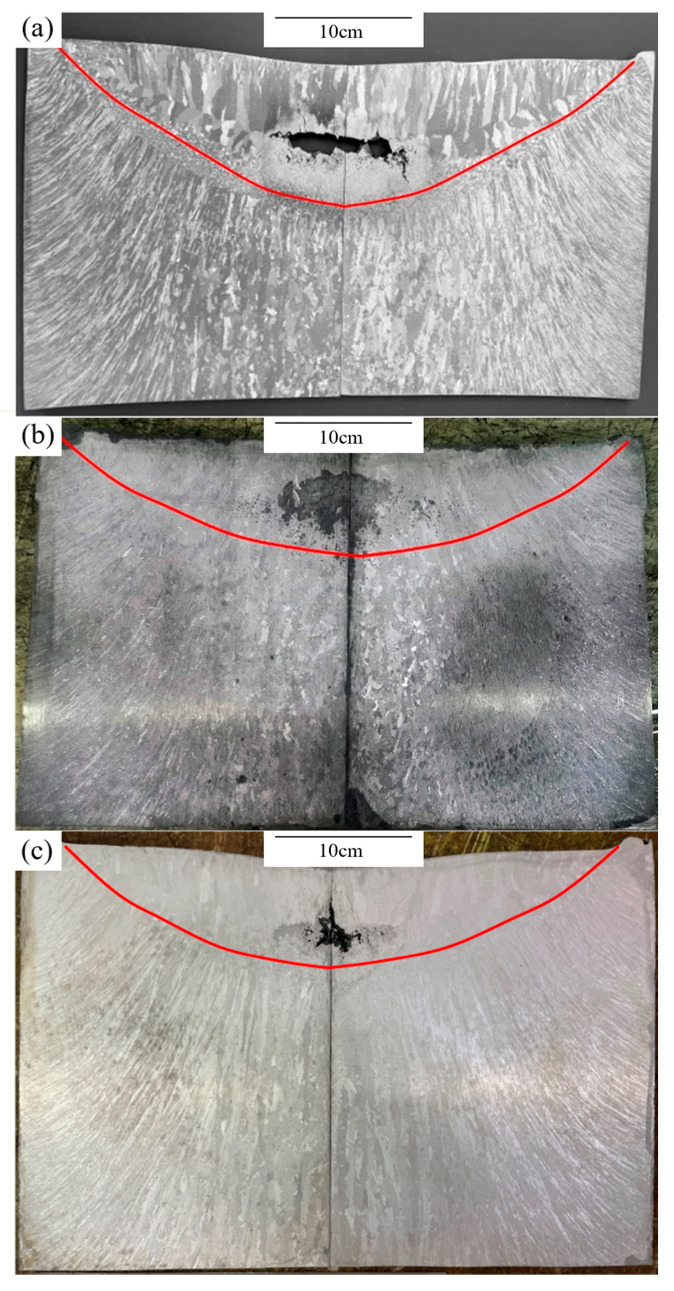
Macrostructure of the IN718 VAR ingots: (**a**) CONSARC furnace (0.48 m/s); (**b**) ALD furnace (0.73 m/s); (**c**) ALD furnace (1.3 m/s). The red line delineates the melt-pool (solid–liquid) interface and was used to measure the melt-pool depth.

In this chapter, the three experimental cooling conditions correspond to three external cooling water flow velocities, i.e., 0.48, 0.73 and 1.30 m/s, which are used as the flow velocity parameters to characterize the external cooling intensity. Figure 7 shows the macrostructures of IN718 alloy Φ 480 mm VAR ingots at different cooling water flow velocities. As shown in Figure 7, the ingots exhibit a typical columnar grain structure throughout, with growth direction inclined from the ingot edge toward the center. Due to water cooling at the external mold wall and helium-enhanced cooling in the ingot solidification gap, the undercooling at the ingot edge is higher, resulting in finer dendrites near the mold wall that gradually coarsen with increasing distance from the mold wall.

At the ingot edge, preferentially oriented columnar dendrite structures can be observed, resulting from crystal preferential growth. Due to convective heat transfer by cooling water on the mold outer wall, a thin layer of fine equiaxed grains forms at the ingot edge under strong convective cooling, which subsequently serves as a nucleation base for columnar grain development. The fine grains at the solidification front gradually grow inward as columnar structures along the heat flow direction, with average grain size and dendrite spacing increasing with distance from the mold wall.

At the end of ingot feeding during melting, the arc at the top extinguishes and heat input is terminated. The molten metal at the ingot top cools under vacuum conditions, and helium gas injection for enhanced cooling is simultaneously stopped. As the melt temperature decreases, the ingot undergoes liquid shrinkage and solidification shrinkage. When the melt at the ingot top surface cools below the solidification point, crystallization proceeds in the form of columnar grains, while enclosed liquid metal regions may form within the ingot. At this stage, if solidification shrinkage cannot be effectively compensated, shrinkage cavities form within the ingot. The comparison in Figure 7 shows that increasing cooling water flow velocity is associated with a reduction in shrinkage cavity size, grain refinement, and a more uniform overall macrostructure. From the molten pool depth curves drawn in Figure 7, cooling water flow velocity has a significant effect on pool morphology and depth. At a flow velocity of 0.48 m/s, the molten pool depth is 117 mm; when increasing it to 0.73 m/s, the pool depth decreases to 88 mm; when the flow velocity further increases to 1.3 m/s, the pool depth decreases to 83 mm. Additionally, at 0.48 m/s flow velocity, the pool shape is U-shaped; at 0.73 m/s, the pool shape is arc-shaped; at 1.3 m/s, the pool shape becomes shallow and flat like a frying pan. The pool symmetry also improves with increasing flow velocity. This trend is consistent with the theoretical analysis in Section 3.2 that increased cooling water flow velocity enhances radial heat transfer and reduces molten pool depth.

### 4.2. Dendrite Microstructure Characteristics of VAR Ingots at Different Cooling Water Flow Velocities

**Figure 8:**
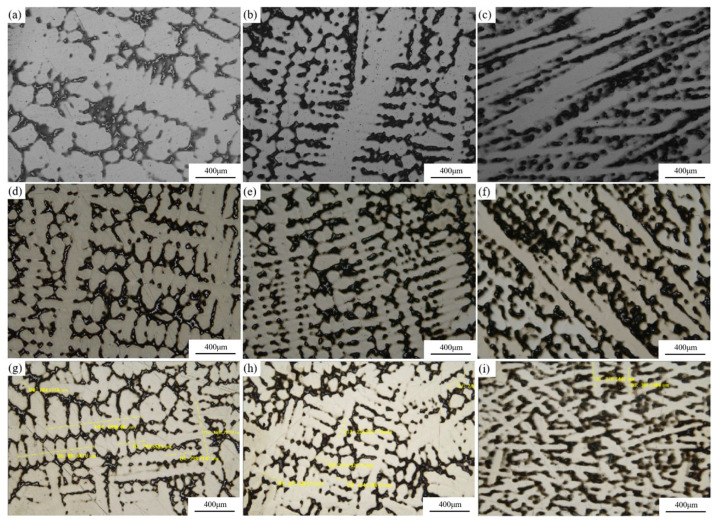
Dendritic structure of the IN718 VAR ingots: (**a**) center at low flow rate; (**b**) R/2 at low flow rate; (**c**) edge at low flow rate; (**d**) center at medium flow rate; (**e**) R/2 at medium flow rate; (**f**) edge at medium flow rate; (**g**) center at high flow rate; (**h**) R/2 at high flow rate; (**i**) edge at high flow rate. The yellow dashed lines are guides for secondary dendrite arm spacing (SADS) measurements; the quantitative SADS results are provided in the text/table.

Typical dendrite morphologies at different flow velocities and positions are shown in Figure 8, with the corresponding secondary dendrite arm spacing (SDAS) values listed in Table 14. As shown in Figure 8, the secondary dendrite arm spacing at the ingot center, which exhibits a lower solidification cooling rate (quantified by the equivalent cooling rate GR in Table 15), is significantly larger than that at the ingot edge with water and helium cooling. With increasing cooling water flow velocity, the secondary dendrite arm spacing at the edge, mid-radius (R/2), and center all decrease significantly, and the degree of elemental segregation is reduced. In addition to the SDAS values, the dendrite morphologies in Figure 8 also show a clear coarsening gradient from the edge to the center under each cooling condition, indicating a longer local solidification time in the inner region. With increasing cooling water flow velocity, the interdendritic network becomes visibly finer at all three radial positions, and the tendency of secondary-arm thickening/fusion observed at the low-flow center is alleviated.

Cooling water flow velocity can affect solidification time by changing solidification rate and temperature gradient. As cooling water flow velocity increases, mold cooling intensity increases, local temperature gradient in the ingot increases, and equivalent cooling rate accelerates, leading to greater undercooling, which is favorable for grain refinement and results in reduced secondary dendrite arm spacing.

From the dendrite morphology perspective, ingots produced at all three flow velocities show dendrite coarsening at the center and R/2 positions. In the ingot center at a cooling water flow velocity of 0.48 m/s, secondary dendrite arms even exhibit mutual fusion. According to metal solidification theory [22], dendrite coarsening is a spontaneous process driven by interfacial free energy. From a thermodynamic perspective, dendrite coarsening reduces the solid–liquid interface area, thereby lowering the total interfacial free energy of the solid–liquid system. The Gibbs–Thomson effect arising from the curvature distribution at the dendrite solid–liquid interface leads to differences in equilibrium composition at the interface, generating concentration gradients in the liquid phase and inducing solute diffusion [23]. These interactions cause melting at high-curvature regions and solidification at low-curvature regions, thereby driving the dendrite coarsening process. The kinetics of this process are controlled by solute transport, while the geometry of the solid–liquid interface directly influences the concentration field evolution during dendrite coarsening. It is generally considered that the degree of coarsening is closely related to local solidification time. Under the same cooling water flow velocity conditions, local solidification time increases from edge to center with decreasing cooling rate, and dendrite coarsening becomes more pronounced. As cooling water flow velocity increases, the cooling rate throughout all ingot regions increases, local solidification time decreases, and the dendrite coarsening process is suppressed. This result is consistent with the analysis in Section 3.2 regarding the enhancement effect of cooling water flow velocity on radial heat transfer.

Quantitatively, when the flow velocity increases from 0.48 to 1.30 m/s, SDAS decreases from 138.2 to 96.4 μm at the center (−30.4%), from 116.1 to 80.4 μm at R/2 (−31.0%), and from 97.6 to 72.4 μm at the edge (−26.5%), confirming that higher external cooling intensity leads to systematic dendrite refinement across the ingot radius (Table 14).

SDAS was quantified from the dendritic micrographs at the center, R/2, and edge using a line intercept approach. For each image, multiple straight intercept lines were placed on well-developed dendritic regions (distributed across the field of view). For each intercept line j, the total measured length $L_{j}$ and the number of secondary-arm spacings $m_{j}$ along that line were recorded, and an individual SDAS value was calculated as $\lambda_{2,j}=L_{j}/m_{j}$. The SDAS reported in Table 14 is the count-weighted mean across all intercept lines, and the scatter is given as one standard deviation. Values are reported as mean ± SD, where n is the total number of spacings counted.

### 4.3. Effect of Cooling Water Flow Velocity on Secondary Dendrite Arm Spacing and Cooling Rate

In this work, the “cooling rate” refers to the ingot solidification cooling rate rather than the water-side cooling rate. Since in situ temperature–time histories were not available, an equivalent cooling rate (GR) was back-calculated from the measured SDAS using an empirical SDAS–GR correlation for IN718; SDAS was measured at the center, R/2 and edge, and the resulting GR values at the three water flow velocities are summarized in Table 15. Secondary dendrite arm spacing (λ_2_) is an important parameter of solidification dendrite structure and serves as a measure of dendrite refinement. Smaller secondary dendrite arm spacing corresponds to finer microstructure, reduced elemental segregation range between dendrite arms, shortened solute element diffusion paths, and reduced diffusion time, thereby lowering the degree of segregation and facilitating homogenization through heat treatment. From a solidification–segregation perspective, IN718 commonly undergoes eutectic-type reactions such as L→γ + NbC and L→γ + Laves, and NbC/Laves are known to precipitate in solute-rich interdendritic liquid. Previous studies have further shown that increasing the cooling rate can inhibit the formation of the Laves phase and mitigate solute enrichment at the solidification front [24]. These reported trends support the present observation that a higher external cooling water flow velocity produces a finer dendritic structure with reduced SDAS, which is generally associated with a reduced segregation length scale and improved chemical homogeneity after subsequent homogenization treatment.

The key factors determining secondary dendrite arm spacing are temperature gradient G and solidification growth rate R. Higher liquid temperature gradient G and faster solidification growth rate R at a given location result in smaller secondary dendrite arm spacing. Based on Fick’s law and the Gibbs–Thomson equation, the relationship between secondary dendrite arm spacing and temperature gradient G and growth rate R can be expressed as [25]:

(18) $$\lambda_{2}=b\times {\left(GR\right)}^{-\beta }$$

where b is an alloy constant.

Based on empirical regression results from existing experimental data, a quantitative relationship between secondary dendrite arm spacing and cooling conditions can be established for IN718 alloy; following the empirical SDAS–cooling rate correlation reported for INCONEL 718 under slow-cooled solidification conditions, the SDAS (λ_2_) and equivalent cooling rate (GR) were related by the expression [24]:

(19) $$\lambda_{2}=258\times {\left(GR\right)}^{-1/3}$$

The equivalent solidification cooling rate GR was back-calculated from SDAS using the empirical SDAS–cooling rate correlation for IN718 (Equation (19)). To retain the measurement statistics, GR was evaluated consistently based on the SDAS measurements from the intercept lines and summarized as a count-weighted mean ± standard deviation using the same n spacings counted in Table 14. The resulting GR values for the three flow velocities at the center, R/2 and edge are listed in Table 15.

The equivalent cooling rate of ingots at different flow velocities was back-calculated from secondary dendrite arm spacing using empirical relationships. The trends are shown in Table 15. When flow velocity increases from 0.48 m/s to 1.3 m/s, the equivalent cooling rate at the center increases by approximately 207.9%, at R/2 by approximately 227.5%, and at the edge by approximately 141.7%, indicating a significant improvement in equivalent cooling rate. Specifically, at v = 0.48 m/s the GR increases from 6.61 K/min at the center to 20.36 K/min at the edge; at v = 0.73 m/s, GR ranges from 13.62 to 44.60 K/min; and at v = 1.30 m/s, GR ranges from 19.26 to 46.56 K/min (Table 15).

### 4.4. Summary of Chapter 4

This chapter systematically investigated the effects of external cooling water flow velocity on the cooling behavior, solidification characteristics, and microstructure evolution of VAR IN718 ingots through macrostructure observation, dendrite characterization, and secondary dendrite arm spacing (SDAS) analysis. The results show that with increasing cooling water flow velocity, the molten pool depth tends to decrease, the pool morphology becomes shallower and more symmetric, and the overall ingot macrostructure exhibits noticeable improvement, indicating enhanced mold cooling effectiveness and improved solidification stability.

At the microstructural level, increasing cooling water flow velocity effectively enhances the solidification cooling intensity at all representative ingot locations and suppresses dendrite coarsening, leading to a systematic reduction in SDAS. Based on the empirical SDAS–cooling rate correlation for IN718, the equivalent solidification cooling rate (GR) was back-calculated from the measured SDAS and was found to increase markedly with flow velocity while exhibiting a clear radial gradient (edge > R/2 > center). These results confirm that cooling water flow velocity is a key processing parameter regulating the solidification conditions and microstructure evolution of VAR ingots within the investigated range. The experimental observations in this chapter are consistent with the theoretical analysis in Section 3.2 regarding the enhancement of radial heat transfer and the improvement of solidification conditions by increasing external cooling water flow velocity, further validating the critical role of cooling water flow velocity in VAR mold cooling regulation.

Regarding chemical composition, the bulk chemistry of the ingots is governed by the feed electrode composition and is not expected to vary with cooling water flow velocity or furnace configuration in the absence of alloy additions; therefore, the “composition variation” relevant to this study primarily refers to dendritic-scale microsegregation controlled by local solidification conditions. Prior work on IN718 has shown that the Nb-rich Laves phase formed by solidification segregation can substantially degrade mechanical performance by acting as a preferred crack initiation/propagation site, and it has been associated with reduced ductility as well as inferior stress rupture and fatigue behavior [26]. Moreover, refinement of the dendritic length scale (i.e., reduced SDAS under higher cooling intensity) has been reported to alleviate interdendritic segregation and suppress Laves–eutectic formation, which is beneficial for subsequent mechanical performance after standard post-solidification thermomechanical/heat treatment routes [27]. In this context, the SDAS/GR trends across different radial positions imply different segregation length scales across the ingot radius. From a property perspective, although hardness measurements were not conducted in the present work, the refined dendritic length scale implied by the decreased SDAS at higher external water flow velocities is generally associated with improved mechanical performance in Alloy 718 cast structures. For investment-cast Alloy 718, SDAS and Vickers hardness have been measured together, and a finer-scale dendritic microstructure has been reported to be expected to exhibit superior mechanical properties [28]. In addition, cooling-rate-controlled cast microstructures in VAR 718 are known to affect subsequent homogenization kinetics and hot-working response through their influence on segregation and second-phase evolution [12]. From a phase perspective, the Nb-rich Laves phase is widely recognized as deleterious: it can act as a preferred crack initiation/propagation site and, by consuming Nb, depletes the matrix of the principal hardening element, thereby reducing ductility and fatigue capability [29]. Therefore, the observed increase in equivalent cooling rate and associated SDAS refinement in the current study is expected to be beneficial for downstream property optimization.

It should be noted that area-resolved bulk chemical analysis and direct characterization of microsegregation/phase constitution (e.g., SEM-BSE/EDS or EPMA mapping, as well as XRD-based phase identification) were not available in the current study due to practical constraints; therefore, discussions regarding segregation constituents and phase formation tendency under different cooling intensities are inferred based on literature-supported solidification mechanisms combined with the experimentally derived cooling rate distribution. Future work will incorporate SEM/EDS (and/or EPMA), XRD, and microhardness mapping/mechanical testing across different radial positions (and, where applicable, tensile/fatigue tests) to quantitatively establish processing–microstructure–property correlations for different configurations and ingot areas.

## 5. Conclusions

1. Based on an equivalent heat transfer model, analysis of VAR mold cooling shows that cooling water flow velocity dominantly enhances overall heat transfer performance during the convective stage, whereas variations in water temperature and specific heat capacity have a markedly weaker effect.
2. The solidification behavior is effectively regulated by cooling water flow velocity, as evidenced by the transition to a shallower, more symmetric molten pool and the systematic refinement of dendrites—SDAS decreases radially from edge to center and is reduced at all locations with increased flow velocity. Specifically, SDAS values decrease by up to 30.4% at the center, 31.0% at R/2, and 26.5% at the edge when the flow velocity increases from 0.48 to 1.30 m/s, confirming that higher external cooling intensity leads to dendritic refinement across the ingot radius.
3. Increased cooling water flow velocity leads to a higher equivalent cooling rate (back-calculated from SDAS), mainly via enhanced radial heat transfer at the helium-filled gap and molten pool, with the R/2 region showing the greatest enhancement. Specifically, the SDAS-derived GR increases from 6.53–18.25 K/min at v = 0.48 m/s, to 13.63–42.38 K/min at v = 0.73 m/s, and further to 19.41–46.01 K/min at v = 1.30 m/s across the center/R/2/edge locations.

## Figures and Tables

**Table 1:** Nominal composition range (UNS N07718) and measured chemical composition of the IN718 electrode used in this work (wt.%).

IN718,UNS N07718	C	Mn	S	P	Cr	Al	Ti
Nominal Range	≤0.08	≤0.35	≤0.015	≤0.015	17.00–21.00	0.20–0.80	0.65–1.15
Measured composition	0.03	0.03	0.003	0.004	19.06	0.44	1.05
IN718,UNS N07718	Mo	Ni	Nb + Ta	B	Co	Fe	
Nominal Range	2.80–3.30	50–55	4.75–5.50	≤0.006	≤1.00	Bal.	
Measured composition	3.06	51.97	5.26	0.005	0.01	Bal.	

**Table 2:** Related parameters of CONSARC furnace.

Parameters of the CONSARC Furnace			
Mold inner diameter	480 mm	Inlet flow rate	3496.4 L/min
Mold wall thickness	25 mm	Inlet temperature	26.2 °C
Water jacket outer diameter	650 mm	Outlet temperature	27.2 °C
Water jacket wall thickness	10 mm	Water jacket height	3000 mm

**Table 3:** VAR experimental conditions under different cooling water flow velocities.

Case No.	VAR Type	Cooling Water Flow Velocity (m/s)	Nominal Ingot Diameter (mm)
Case 1	CONSARC	0.48	480
Case 2	ALD	0.73	480
Case 3	ALD	1.30	480

**Table 4:** Thermal resistance allocation in non-helium gap sections.

Heat Transfer Stage	Thermal Resistance (m^2^·K/W)	Proportion (%)
Radiative heat transfer from ingot to mold inner wall	0.121	99.50
Conduction through mold wall	6.76 × 10^−5^	0.06
Convective heat transfer at mold outer wall to cooling water	5.44 × 10^−4^	0.44

**Table 5:** Optimization effect of flow velocity on heat transfer coefficient in helium-free gap.

Flow Velocity v (m/s)	Convective Heat Transfer Coefficient h_2a_ (W/(m^2^·K))	Overall Heat Transfer Coefficient h_a_ (W/(m^2^·K))	Improvement (%)
0.48	1839.6	8.223	\
0.70	2486.3	8.232	1.1
0.90	3039.9	8.237	1.7
1.10	3569.3	8.241	2.2
1.30	4079.7	8.243	2.4
2.00	5758.3	8.248	3.0
3.00	7964.7	8.251	3.4
4.00	10,025.9	8.253	3.6

**Table 6:** Proportion of thermal resistance contributions from each component in the helium-filled gap.

Heat Transfer Stage	Thermal Resistance (m^2^·K/W)	Proportion (%)
Helium convective heat transfer to mold inner wall	7.13 × 10^−4^	53.83
Conduction through mold wall	6.76 × 10^−5^	5.10
Convective heat transfer at mold outer wall to cooling water	5.44 × 10^−4^	41.07

**Table 7:** Optimization effect of flow velocity on heat transfer coefficient in helium-filled gap.

Flow Velocity v (m/s)	Convective Heat Transfer Coefficient h_2b_ (W/(m^2^·K))	Overall Heat Transfer Coefficient h_b_ (W/(m^2^·K))	Improvement (%)
0.48	1839.6	754.94	\
0.70	2486.3	845.45	12.0
0.90	3039.9	901.23	19.4
1.10	3569.3	942.86	24.9
1.30	4079.7	974.94	29.1
2.00	5758.3	1047.89	38.8
3.00	7964.7	1103.51	46.2
4.00	10,025.9	1135.98	50.5

**Table 8:** Proportion of thermal resistance contributions from each component in the molten pool.

Heat Transfer Stage	Thermal Resistance (m^2^·K/W)	Proportion (%)
Heat transfer through solidified shell	2 × 10^−4^	24.64
Conduction through mold wall	6.76 × 10^−5^	8.33
Convective heat transfer at mold outer wall to cooling water	5.44 × 10^−4^	67.03

**Table 9:** Optimization effect of flow velocity on heat transfer coefficient in the molten pool.

Flow Velocity v (m/s)	Convective Heat Transfer Coefficient h_2_c (W/(m^2^·K))	Overall Heat Transfer Coefficient h_c_ (W/(m^2^·K))	Improvement (%)
0.48	1839.6	1232.13	\
0.70	2486.3	1492.98	21.2
0.90	3039.9	1676.16	36.0
1.10	3569.3	1825.82	48.2
1.30	4079.7	1950.46	58.3
2.00	5758.3	2266.03	83.9
3.00	7964.7	2543.56	106.4
4.00	10,025.9	2722.57	121.0

**Table 10:** Relationship between bottom distance and heat transfer coefficient in the molten pool.

Distance from Pool to Bottom (mm)	Overall Heat Transfer Coefficient h_d_ (W/(m^2^·K))
5	2127.66
20	934.58
100	234.19
200	120.92
1000	24.83
2000	12.46

**Table 11:** Water viscosity at various temperatures.

Temperature t_f_ (°C)	Water Viscosity μ (Pa·s)
0	1.792 × 10^−3^
10	1.308 × 10^−3^
20	1.002 × 10^−3^
30	7.970 × 10^−4^
70	4.040 × 10^−4^
110	2.400 × 10^−4^

**Table 12:** Optimization effect of temperatures in gap and molten pool on total heat transfer coefficient.

Water Temperature (°C)	Location	Convective Heat Transfer Coefficient h_2b_, h_2c_ (W/(m^2^·K))	Overall Heat Transfer Coefficient h_b_, h_c_ (W/(m^2^·K))	Improvement (%)
30	Gap	1982.4	781.25	\
	Molten pool	2099.2	1344.09	\
20	Gap	2032.9	785.85	0.59
	Molten pool	2152.8	1365.93	1.62
10	Gap	2093.4	794.72	1.72
	Molten pool	2216.8	1391.40	3.52
0	Gap	2167.1	805.13	3.06
	Molten pool	2294.9	1421.87	5.79

Note: “Gap” corresponds to the He-filled gap stage (Section 3.2.2), and “Molten pool” corresponds to the molten pool radial stage (Section 3.2.3). Accordingly, the water-side convection coefficient is denoted as $h_{2b}$ (Gap) and $h_{2c}$ (Molten pool), and the overall coefficient is $h_{b}$ (Gap) and $h_{c}$ (Molten pool).

**Table 13:** Optimization effect of specific heat capacities in gap and molten pool on total heat transfer coefficient.

Specific Heat Capacity c (J/(kg·K))	Location	Convective Heat Transfer Coefficient h_2b_, h_2c_ (W/(m^2^·K))	Overall Heat Transfer Coefficient h_b_, h_c_ (W/(m^2^·K))	Improvement (%)
4.18 × 10^3^	Gap	1839.6	754.94	\
	Molten pool	1839.6	1232.13	\
4.4 × 10^3^	Gap	1878.1	761.56	0.88
	Molten pool	1878.1	1249.84	1.44
4.6 × 10^3^	Gap	1911.8	767.04	1.60
	Molten pool	1911.8	1264.70	2.64
4.8 × 10^3^	Gap	1944.6	772.32	2.30
	Molten pool	1944.6	1279.10	3.81

Note: “Gap” corresponds to the He-filled gap stage (Section 3.2.2), and “Molten pool” corresponds to the molten pool radial stage (Section 3.2.3). Accordingly, the water-side convection coefficient is denoted as $h_{2b}$ (Gap) and $h_{2c}$ (Molten pool), and the overall coefficient is $h_{b}$ (Gap) and $h_{c}$ (Molten pool).

**Table 14:** Secondary dendrite arm spacing of the IN718 VAR ingots.

Secondary Dendrite Arm Spacing λ_2_ (μm)	CONSARC Furnace (Flow Velocity 0.48 m/s)	ALD Furnace (Flow Velocity 0.73 m/s)	ALD Furnace (Flow Velocity 1.3 m/s)
Center	138.2 ± 7.2 (n = 25)	108.2 ± 3.1 (n = 28)	96.4 ± 3.2 (n = 19)
R/2	116.1 ± 2.9 (n = 33)	85.4 ± 1.7 (n = 36)	80.4 ± 1.1 (n = 30)
Edge	97.6 ± 7.3 (n = 31)	73.5 ± 5.7 (n = 16)	72.4 ± 5.1 (n = 24)

**Table 15:** Equivalent solidification cooling rates (GR) of IN718 VAR ingots at different external water flow velocities.

Furnace	Flow Velocity v (m/s)	Secondary Dendrite Arm Spacing λ_2_ (μm)	Equivalent Cooling Rate GR (K/min)	Improvement in Equivalent Cooling Rate
CONSARC	0.48	Center: 138	6.61 ± 1.02 (n = 25)	\
		R/2: 116	13.62 ± 1.24 (n = 28)	
		Edge: 98	19.26 ± 1.87 (n = 19)	
ALD	0.73	Center: 108	11.00 ± 0.83 (n = 33)	Center: 66.4
		R/2: 85	27.68 ± 1.66 (n = 36)	R/2: 103.2
		Edge: 74	33.06 ± 1.34 (n = 30)	Edge: 71.6
ALD	1.30	Center: 96	20.36 ± 4.69 (n = 31)	Center: 207.9
		R/2: 80	44.60 ± 9.39 (n = 16)	R/2: 227.5
		Edge: 72	46.56 ± 9.27 (n = 24)	Edge: 141.7

## Data Availability

The original contributions presented in this study are included in the article. Further inquiries can be directed to the corresponding author.
